# Variability in Doctors’ Usage Paths of Mobile Electronic Health Records Across Specialties: Comprehensive Analysis of Log Data

**DOI:** 10.2196/12041

**Published:** 2019-01-17

**Authors:** Ji Yeong Soh, Sang-Hyuk Jung, Won Chul Cha, Mira Kang, Dong Kyung Chang, Jaegon Jung, JeanHyoung Lee, Jong Soo Choi, Kyunga Kim

**Affiliations:** 1 Department of Digital Health Samsung Advanced Institute for Health Sciences & Technology Sungkyunkwan University Seoul Republic of Korea; 2 Department of Emergency Medicine Samsung Medical Center Sungkyunkwan University School of Medicine Seoul Republic of Korea; 3 Health Information and Strategy Center Samsung Medical Center Seoul Republic of Korea; 4 Center for Health Promotion Samsung Medical Center Sungkyunkwan University School of Medicine Seoul Republic of Korea; 5 Department of Gastroenterology Samsung Medical Center Sungkyunkwan University School of Medicine Seoul Republic of Korea; 6 Department of Computer Engineering Seoul Digital University Seoul Republic of Korea; 7 Statistics and Data Center Research Institute for Future Medicine Samsung Medical Center Seoul Republic of Korea

**Keywords:** mobile apps, electronic health record, mobile health, mobile electronic health record

## Abstract

**Background:**

With the emergence of mobile devices, mobile electronic health record (mEHR) systems have been utilized by health care professionals (HCPs), including doctors, nurses, and other practitioners, to improve efficiency at the point of care. Although several studies on mEHR systems were conducted, including those analyzing their effects and HCPs’ usage frequency, only a few considered the specific workflows of doctors based on their specialties in which the work process differs greatly.

**Objective:**

This study aimed to investigate the differences in mEHR usage paths across clinical specialties.

**Methods:**

We collected the log data of 974 doctors who worked from August 2016 to August 2017 and used the mEHR system at the Samsung Medical Center, one of the biggest hospitals in South Korea. The doctors were classified into 3 groups based on their specialty: the physician, the surgeon, and other hospital-based physician (OHBP) groups. We used various descriptive and visualization methods to understand and compare doctors’ usage paths of mEHRs. First, the average numbers of log-ins per day and features used per log-in were examined over different specialties and positions. Second, the number of features used by each doctor was visualized via a heat map to provide an overview of mEHR usage across feature types and doctors’ specialties. Third, we conducted a path analysis via a Sankey diagram to describe main usage paths and association rule mining to find frequent paths in mEHR usage.

**Results:**

The physician group logged on most frequently, whereas the OHBP group logged on least frequently. In fact, the number of log-ins per day of residents in the physician group was 4.4 times higher than that of staff members in the other groups. The heat map visualization showed a visible difference among specialty groups. The physician group used more consultation-related features, whereas the surgeon group used more surgery-related features. Generally, 50% of the doctors spent about 15 seconds at a time when using mEHRs. In the Sankey diagram, the physician group showed diverse usage patterns with higher complexity compared with the other 2 groups; in particular, their paths contained more loops, which reflected repetitive checks on multiple patients. The most frequent path included inpatient summary, which means that most users stopped at the point of summary and did not proceed to view more details.

**Conclusions:**

The usage paths of mEHRs showed considerable differences among the specialty groups. Such differences can be accommodated into an mEHR design to enhance the efficiency of care.

## Introduction

### Background

The advent of mobile phones has accelerated the expansion of mobile health (mHealth) market because they are equipped with various apps and functions such as wireless connectivity and messaging capabilities. Mobile phone–based mHealth apps have emerged as strong tools for patients and health care professionals (HCPs) in the digital health care era [1,2]. The development of mHealth apps for the private market has been promoted as a result of increasing global access to mobile technology [3].

Many institutions seek mobile electronic health record (mEHR) systems that can improve efficiency at the point of care [4]. The mEHRs provide HCPs with ubiquitous access to patient data in real time, and hence, enable them to communicate with others when facilitating a patient’s care [5-10]. A well-made mEHR can improve workflow efficiency, thereby lowering costs and reducing the work burden of HCPs [11,12]. Although the overall satisfaction rate of mEHRs has increased [11,12], their benefits and satisfaction may differ among doctors (ie, medical doctors), according to the latter’s specialties [13].

Recently, efforts have been made to analyze log data from mEHRs for the evaluation of providers’ workflow [14,15]. Even among doctors, the usage paths are likely to differ according to their specialties, resulting in different work processes [13]. However, research on specialty-based paths with an in-depth analysis has not yet been conducted. This is insufficient to reflect the characteristics of its practical use.

### Objectives

In this study, we analyzed real mEHR log data of doctors and investigated specialty-based mEHR usage paths. The difference in the usage paths can be reflected in mEHRs to improve their efficiency and usability.

## Methods

### Mobile Electronic Health Record System

**Figure 1:**
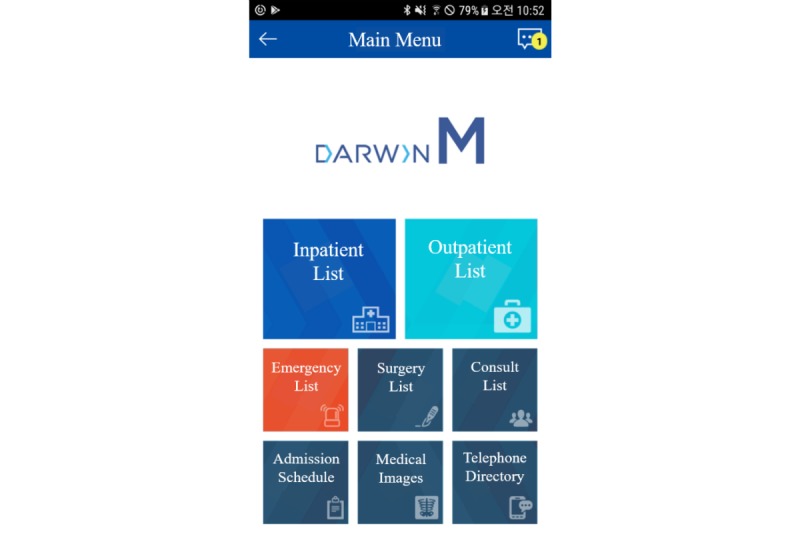
A screenshot of the mDARWIN screen displayed after login. The main menu functions as a portal for specific contents. mDARWIN: Data Analysis & Research Window for Integrated kNowledge.

**Figure 2:**
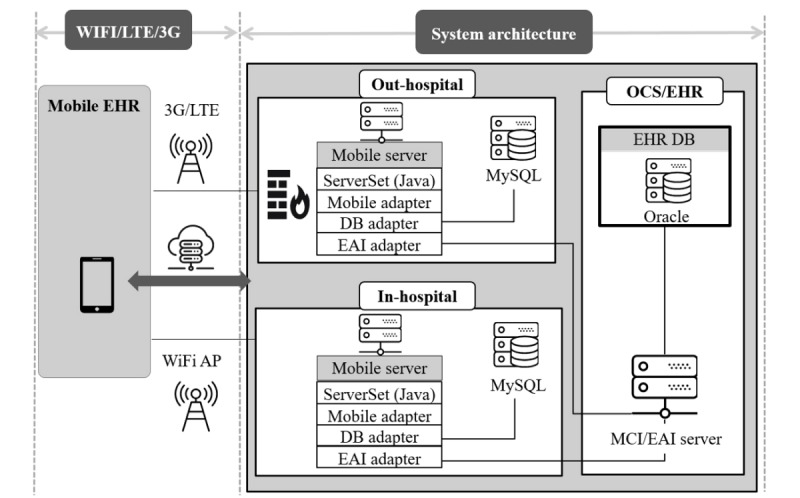
System architecture of mDARWIN. It was designed to accommodate 2 different network connectivity choices. AP: access point; DB: database; EAI: enterprise application integration; EHR: electronic health record; LTE: long-term evolution; MCI: multi-channel integration; OCS: order communication system; SQL: structured query language.

**Figure 3:**
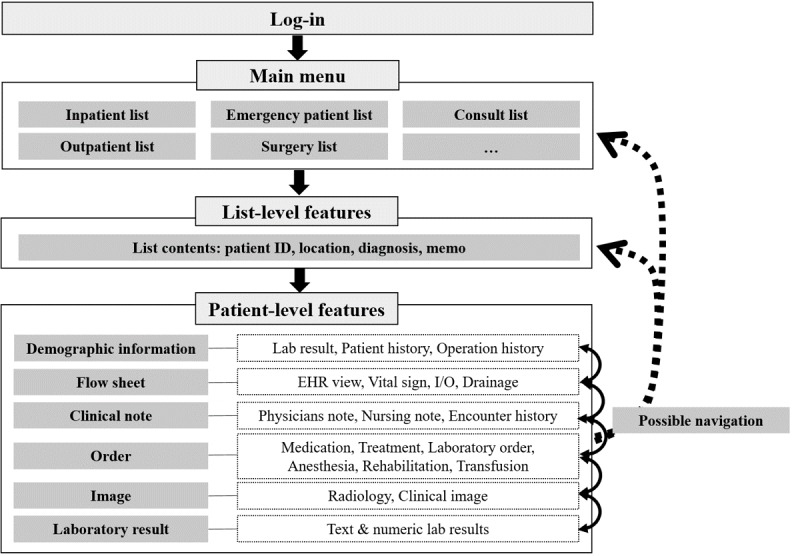
Feature list of mDARWIN. The system consists of 3 levels: main menu, list-, and patient-level features. EHR: electronic health record; ID: identification; I/O: input/output.

This study was conducted at the Samsung Medical Center (SMC), one of the largest tertiary referral hospitals in South Korea with more than 2000 beds and approximately 1000 doctors. In 2017, the average daily visit was about 8000 and 220 for the outpatient and emergency departments, respectively. The next-generation medical information system, including a new version of an electronic health record (EHR) system known as the Data Analysis & Research Window for Integrated kNowledge (DARWIN), was launched in July 2016. At the same time, the previous mEHR system was majorly revised and launched with a new name, mDARWIN version 2.3.7-2.4.8 (Figure 1). mDARWIN is based on Android 2.3 Gingerbread (Google Inc, California, United States) and has Wi-Fi and 3G capabilities (Figure 2). It comprises a main menu, list-level features, and patient-level features (Figure 3). The app is mainly for the use of doctors. After log-in, on using a certified user’s identification number and password, users can choose from the main menu to view a list-level feature or select a function. From each list-level feature, users can choose patient-level features for more activities or leave and move to other list-level features. Each session closes when either a user logs out or it automatically logs out after no activity for a certain amount of time. mDARWIN also supports fingerprint log-in and near-field communication.

### Study Subjects and Data Collection

Target subjects were doctors who had logged on to the mEHR system from August 2016 to August 2017. Visiting doctors were excluded because of short usage duration. Doctors who used the system at least once a month were still included in the analysis. To examine the association between usage and specialty, the subjects were categorized into 3 groups based on their specialties. The physician group consisted of internal medicine, family medicine, pediatrics, and critical care. The surgeon group included general surgery, neurosurgery, and otorhinolaryngology. The other hospital-based physician (OHBP) group covered anesthesiology, pathology, and radiology. The subjects were further classified by job position (staff members, clinical fellows, and residents). The log data for all subjects were collected from the mDARWIN server. For each subject, sessions were identified as a series of features used from log-in to logout. The sessions lasting longer than 60 min were filtered out. This study was approved by the institutional review board of the study site (SMC 2017-12-074).

### Data Analysis

Overall usage for individual features in the mEHR system was investigated by summarizing usage frequencies of features from the log data. The frequencies were normalized within each specialty group and presented against specialty departments in a heat map visualization [16]. For each specialty group, usage paths were identified in 3 steps. First, all pairs of adjacent features in every session were recognized. Second, we computed the amount of the first-order transition for each feature pair.

Finally, usage paths were constructed as sets of feature pairs with large first-order transition amounts for each specialty group and then visualized using Sankey diagrams [17]. For better visualization, flows with small frequencies were omitted from the diagrams. In addition, we performed association rule mining (ARM) to identify the top 5 usage paths per specialty according to support values [18].

All analyses were performed using R software version 3.4.3 (R Foundation for Statistical Computing, Vienna, Austria) [19].

## Results

### Principal Results

**Figure 4:**
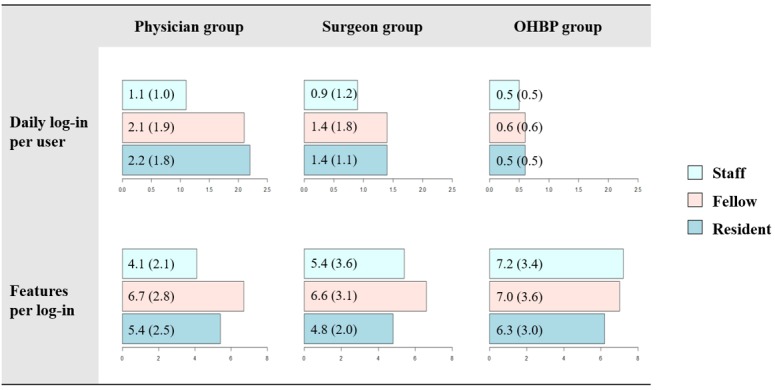
The average numbers of daily log-ins per user and features per log-in according to users’ specialty and position. The numbers in parentheses stand for standard deviation. OHBP: other hospital-based physician.

**Figure 5:**
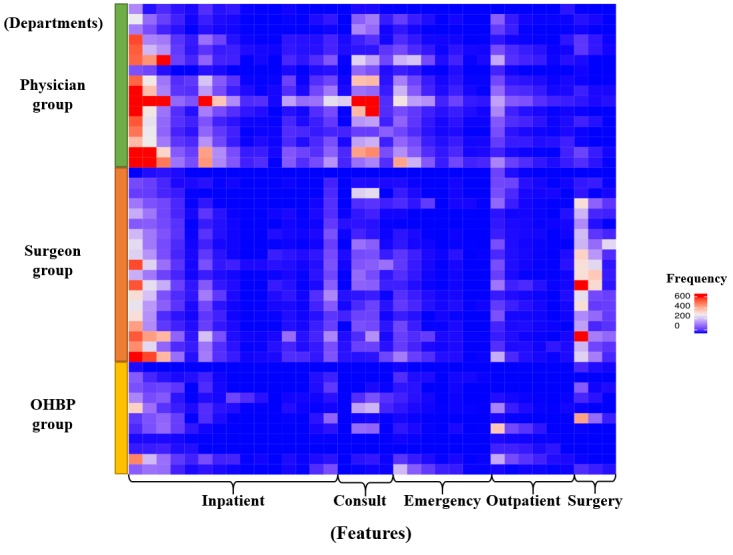
Heat map visualization of feature usage patterns according to users’ specialties. Rows and columns stand for specialty departments and individual features, respectively. OHBP: other hospital-based physician.

During the study period, 974 unique doctors used mEHRs and generated 2,777,311 event logs, with the following distribution: the physician group, 46.9% (457/974); the surgeon group, 39.4% (384/974); and the OHBP group, 13.7% (133/974). The average number of daily log-ins per user and content per log-in by specialty group and position are shown in Multimedia Appendix 1. There were 24.8% (242/974) staff members, 23.7% (231/974) clinical fellows, and 51.4% (501/974) residents. The average number of daily log-ins per user was 1.4, with an SD of 1.5. After each log-in, users visited 5.5 features (SD 2.8) on average. Doctors in the physician group, especially residents, showed the most frequent log-in activities (2.2 and 3 times more frequent than the surgeon and OHBP groups, respectively). Doctors in the OHBP group, especially staff members, tended to visit diverse features per log-in, compared with the other groups (see Figure 4). Different usage of features was observed among specialties in the heat map (Figure 5). Frequently used features were indicated as hotspots in the heat map and differed across users’ specialties. There were hotspots in the consultation-related features for the physician group and in the surgery-related features for the surgeon group. The OHBP group used features evenly, among most features, whereas the other groups used a specific set of features intensively. No distinguished difference was observed in the use of emergency- and outpatient-related features for all 3 groups. Compared with other features, the usage of outpatient features was less frequent in all 3 groups.

### Path Analysis

**Figure 6:**
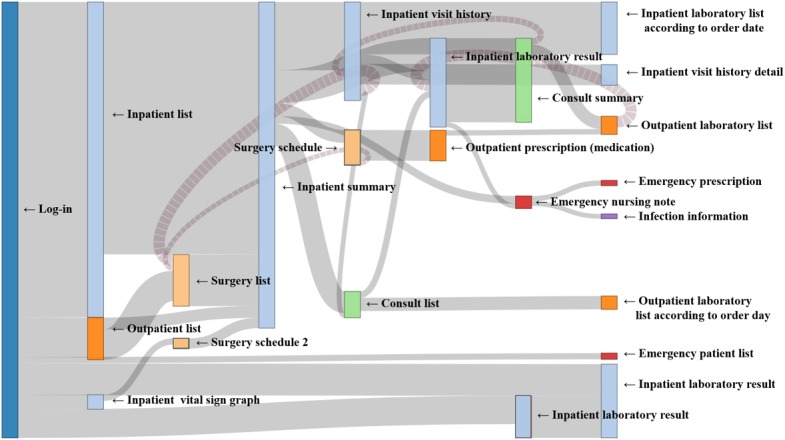
Sankey diagram of usage paths identified for the Physician group. Light blue, light green, light orange, orange and red colors were used to indicate impatient, consult, surgery, outpatient and emergency features, respectively.

**Figure 7:**
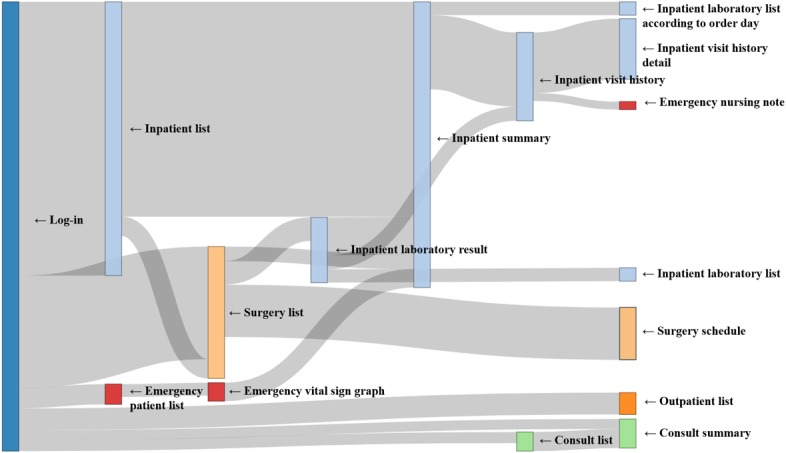
Sankey diagram of usage paths identified for the Surgeon group. Light blue, light green, light orange, orange and red colors were used to indicate impatient, consult, surgery, outpatient and emergency features, respectively.

**Figure 8:**
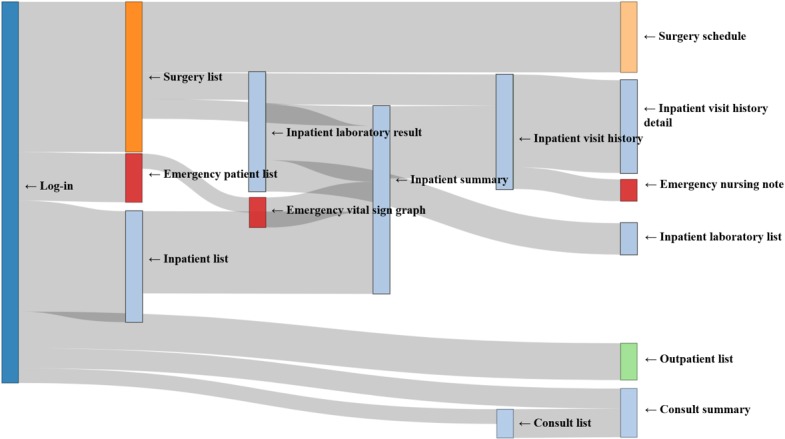
Sankey diagram of usage paths identified for the other hospital-based physicians (OHBP) group. Light blue, light green, light orange, orange and red colors were used to indicate impatient, consult, surgery, outpatient and emergency features, respectively.

The identified usage paths were specialty-specific, in that they varied across specialties (see Sankey diagrams in Figures 6-8). Compared with the other 2 groups showing heavy flows to surgical features, the physician group showed diverse flows and paths. For instance, they showed repetitive transition patterns among the same features (often called loops), whereas the surgeon and the OHBP groups did not form loops and had more simple paths. The repetitive patterns seemed to reflect physicians’ work processes containing repetitive checks on multiple patients.

Among the top 5 paths identified via ARM for each group, most paths included an *inpatient summary* feature, with a high support value of more than 40% (Table 1) [20]. However, the 2-feature path from *inpatient list* to *inpatient summary* was most frequently taken than multiple-feature paths. This finding implied that most users tended to stop at the point of summary and did not proceed to view more details. Regarding frequently used paths, consultation- and emergency-related paths were recognized in the physician group, whereas the operation-related path was identified in the surgeon and the OHBP groups. For all 3 groups, outpatient features were not ranked in the top 5 paths.

**Table 1:** Top 5 usage paths identified via association rule mining for each specialty group.

Specialty, rank and path^a^		N	Support (%)	Confidence (%)	Lift
**Physician group**					
	Inpatient list (77.59) → inpatient summary (54.59)	98,590	47.93	61.77	1.13
	Consult list (12.48) → consult summary (12.81)	17,594	8.55	66.79	5.35
	Inpatient list (77.59) → inpatient summary (54.59) → inpatient visit history (8.5300)	16,594	8.07	14.78	1.73
	Emergency patient list (12.86) → emergency vital sign graph (7.11)	14,614	7.10	55.22	7.77
	Inpatient list (77.59) → inpatient summary (54.59) → inpatient visit history (8.53) → inpatient visit history detail (4.08)	8348	4.06	47.56	11.66
**Surgeon group**					
	Inpatient list (64.61) → inpatient summary (50.36)	45,777	40.54	80.50	1.25
	Inpatient list (64.61) → inpatient summary (50.36) → inpatient visit history (10.63)	11,886	10.53	20.90	1.97
	Inpatient list (64.61) → inpatient summary (50.36) → inpatient laboratory result (8.76)	9328	82.61	16.40	1.98
	Operation list (29.23) → surgery schedule (8.12)	9056	80.20	27.44	3.38
	Inpatient list (64.61) → inpatient summary (50.36) → inpatient visit history (10.63) → inpatient visit history detail (6.27)	7058	6.25	58.78	9.37
**Other hospital-based physician group**					
	Inpatient list (32.69) → inpatient summary (40.46) → inpatient visit history (18.84)	3200	18.68	46.16	2.45
	Inpatient list (32.69) → inpatient summary (40.46)	3123	18.23	45.05	1.38
	Inpatient list (32.69) → inpatient summary (40.46) → inpatient laboratory result (15.87)	2719	15.87	39.22	2.47
	Operation list (40.25) → surgery schedule (14.81)	2532	14.78	36.72	2.48
	Inpatient list (32.69) → inpatient summary (40.46) → inpatient visit history (18.84) → inpatient visit history detail (12.22)	2263	13.21	70.13	5.31

^a^Features in each path are listed with their support (ie, usage ratio per session) in parentheses.

## Discussion

### Principal Findings

Evidence for the effectiveness of mobile apps on health care is increasing [21]. EHRs are also viewed as a means of improving HCPs’ decisions and clinical health outcomes [22]. However, the level of evidence on the value of mEHR is relatively low. Analyzing the work process of users, which can be matched later to clinical implication, is necessary to measure the value of any health information technology (IT) system [23].

For the users of IT systems, including mEHRs, it is essential to acquire appropriate information with the least number of click-throughs, such as log-ins, transitions, and navigation. The types and amount of information must be more tailored and intuitively visualized for the user’s intent [24]. If IT solutions are not refined enough, they would increase the burden on the workflow [23]. The problem lies in the fact that HCPs do not have the answer to optimization before using them in the field or even after using them for a while. Therefore, the understanding of current usage patterns is a crucial part in system refinement and optimization.

In this study, we conducted a comprehensive analysis of the mEHR log data to investigate doctors’ usage patterns using multiple analytic tools, such as a heat map, a Sankey diagram, and ARM. The heat map showed a cross-sectional volumetric view of the association between users and services (ie, departments and individual features) and hence enabled us to examine overall usage patterns of individual features according to the specialty. The Sankey diagram used the information on the first-order transition between 2 features and presented the sequential characteristics of usage patterns. Examples include frequent transitions, repetitive visits, and loops. ARM assessed co-occurrence of 2 or multiple features with quantitative criteria and identified important paths by searching for frequent if-then relationships among features. These criteria, such as support, confidence, and lift, helped to further characterize the identified paths. Our comprehensive analytic approach can be a good starting point to understand the current usage status of an mEHR and hence reveal direction for better usability (eg, feature development and user-interface modification).

More frequent use of the mEHR was observed for the physician group than for the other groups. In the volumetric view and sequential characteristic analysis, physicians tended to utilize more inpatient features and navigate through multiple low-level features in a repetitive manner. These observations implied that the current mEHR environment is more targeted at physicians who need to look up the system as they conduct inpatient care and daily patient rounds across different locations. Therefore, some improvement can be pursued to make repetitive transitions among frequently used features more efficient.

Compared with physicians, surgeons and OHBPs connected the system less frequently and used a smaller number of features. It is partly due to the shortage of specialty-specific features for them. Surgeons, especially, may benefit from features or tools related to the operating theater. For instance, augmented reality and virtual reality technology focused on the surgery process would be points of improvement for surgical specialties.

Outpatient features showed a very low usage rate in all specialty groups. It seems natural in that a desktop-based system might be more effective where doctors do not need to move around (eg, medical office, examination, and consultation rooms). To improve system efficiency, the mEHR can be modified by removing never-used features and changing the order of appearance of features according to their usage frequencies and so on.

### Limitations

First, this is a single-system study with in-house software, which could bear a potential limitation for generalization. However, mEHR systems in most institutes are in the developing status, and no sufficient consensus over its standard is reached. This single-system analysis is still valuable in terms of evidence.

Second, the outcomes and measurements of this study were set only on mobile logs. Neither the practical and clinical purpose nor subjective opinions by doctors were considered. When an in-depth log analysis is combined with an investigation of users’ perception, the usability of an mEHR system can be comprehensively evaluated. This comprehensive evaluation can lead to connecting the need of electronic features to clinical process and, thereby, to better system development.

Third, the offline characteristics of the specific department that utilized the features were not reflected. The difference of mEHR and EHR utilization patterns was not considered, which limits the interpretation of results on practical practices.

### Conclusions

In this study, a comprehensive analysis of the mEHR log data revealed considerable differences in usage patterns among specialty groups of medical doctors. The usage paths were further characterized for each specialty and demonstrated the need and direction for the improvement of the current system including specialty-specific user interfaces.

## Abbreviations

ARM: association rule mining
DARWIN: Data Analysis & Research Window for Integrated kNowledge
EHR: electronic health record
HCP: health care professional
IT: information technology
mEHR: mobile electronic health record
mHealth: mobile health
NRF: National Research Foundation of Korea
OHBP: other hospital-based physician
SMC: Samsung Medical Center

## Appendix

Multimedia Appendix 1 Average number of daily log-ins per user and content per log-in by specialty group and position.
