# Supplementary material for: Variability in Doctors’ Usage Paths of Mobile Electronic Health Records Across Specialties: Comprehensive Analysis of Log Data
Source: JMIR Mhealth Uhealth. 2019 Jan 17;7(1):e12041. doi: 10.2196/12041 (PMC6354233; doi:10.2196/12041)
Supplement: Multimedia Appendix 1 [file mhealth_v7i1e12041_app1.pdf]

Appendix 1. Average number of daily logins per user and content per login by specialty group and position

**Position**		**Physician group (N = 457)**	**Surgeon group (N = 384)**	**OHBP group (N = 133)**	**Total (N = 974)**
**Staff (N = 242)**	Logins per day/person	1.1 (±1.0)	0.9 (±1.2)	0.5 (±0.5)	0.9 (±1.0)
	Contents per login	4.1 (±2.1)	5.4 (±3.6)	7.2 (±3.4)	4.9 (±3.0)
**Fellow (N = 231)**	Logins per day/person	2.1 (±1.9)	1.4 (±1.8)	0.6 (±0.6)	1.7 (±1.8)
	Contents per login	6.7 (±2.8)	6.6 (±3.1)	7.0 (±3.6)	6.7 (±3.0)
**Resident (N = 501)**	Logins per day	2.2 (±1.8)	1.4 (±1.1)	0.6 (±0.6)	1.6 (±1.5)
	Contents per login	5.4 (±2.5)	4.8 (±2.0)	6.3 (±3.0)	5.3 (±2.5)
**Total (N = 974)**	Logins per day/person	1.8 (±1.7)	1.3 (±1.3)	0.6 (±0.6)	1.4 (±1.5)
	Contents per login	5.4 (±2.6)	5.3 (±2.7)	6.6 (±3.2)	5.5 (±2.8)
